# A Systematic Review of Sacral Insufficiency Fractures: Treatment Modalities and Outcomes

**DOI:** 10.7759/cureus.41745

**Published:** 2023-07-11

**Authors:** Praise Briggs, Samuel W King, Tim Staniland, Shivkumar Gopal, Rajesh Shah, Mukai Chimutengwende-Gordon

**Affiliations:** 1 Trauma and Orthopaedics, Hull University Teaching Hospitals NHS Trust, Kingston upon Hull, GBR; 2 Trauma and Orthopaedics, Bradford Teaching Hospitals NHS Foundation Trust, Bradford, GBR; 3 Knowledge and Library Services, Hull University Teaching Hospitals NHS Trust, Kingston upon Hull, GBR; 4 Pelvic and Hip Orthopaedic Surgery, Hull University Teaching Hospitals NHS Trust, Kingston upon Hull, GBR; 5 Pelvic and Spinal Orthopaedic Surgery, Hull University Teaching Hospitals NHS Trust, Kingston upon Hull, GBR; 6 Pelvic Orthopaedic Surgery, Hull University Teaching Hospitals NHS Trust, Kingston upon Hull, GBR

**Keywords:** systematic review, outcomes, conservative management, screw fixation, surgical fixation, sacral insufficiency fracture, sacroplasty

## Abstract

Sacral insufficiency fractures can be managed conservatively, by surgical fixation or by sacroplasty. This systematic review compared the outcomes of different treatment modalities to ascertain the best. Studies included in this systematic review were those with sacral insufficiency fracture in elderly patients with some measure of outcomes reported. Fractures due to high-energy trauma or malignancy or in non-elderly patients were excluded. The review was carried out according to the Preferred Reporting Items for Systematic Reviews and Meta-Analyses (PRISMA) guidelines. MEDLINE and Embase databases were searched from inception to January 6, 2022, yielding a total of 4299 papers of which 35 were eligible for inclusion. Pain reduction following sacroplasty (Visual Analogue Scale (VAS) difference 5.83, SD 1.14, n = 901) was superior compared with conservative management (VAS difference 3.7, SD 2.71, n = 65) (p <0.0001) and surgical fixation (with screws/rods +/- cement augmentation; VAS difference 4.1, SD 1.106, n = 154) (p< 0.001). There was no significant difference between pain relief following screw fixation and after conservative management (p = 0.1216). Hospital stay duration was shorter following sacroplasty (4.1 days )SD 3.9)) versus fixation (10.3 days (SD 5.59)) (p= 0.0001). Available evidence points to sacroplasty resulting in better pain relief and shorter hospital stay than other treatment options.

## Introduction and background

Sacral insufficiency fractures (SIF) are stress fractures of the sacrum, associated with osteoporosis and are common in the elderly population [1-7]. Treatments can be defined in three categories: conservative, sacroplasty, and surgical fixation [1]. While conservative treatment with bed rest, analgesics, and medications to treat osteoporosis has yielded some positive results, it is often complicated by problems of prolonged immobility such as atelectasis, pressure ulcers, deep vein thrombosis, pulmonary embolism, loss of mobility, and debilitating pain [1]. Although initial clinical improvement occurs rapidly, complete resolution may not occur for as long as 9-12 months [8]. Meanwhile, studies have shown an improvement in pain scores and mobility for patients who have surgical fixation of SIF [9-11]. However, there are concerns about bone quality and screw hold in osteoporotic bone [12]. Sacroplasty involves injection of polymethylmethacrylate (PMMA) bone cement into the sacrum and has been shown to have good pain relief and early return to mobility [3,8] but there are concerns about the effects of cement extravasation, especially around nerve roots [5,9]. This systematic review aims to ascertain the existing evidence for treatment options, and whether good quality evidence is available to provide the basis for guidelines for treatment of SIF.

## Review

Methods

The review process was conducted according to the Preferred Reporting Items for Systematic Reviews and Meta-Analysis (PRISMA) guidelines. A preliminary search was undertaken using Healthcare Databases Advanced Search (HDAS) to identify suitable search terms and create the following PICO (population, intervention, control, and outcomes) search strategy [13]: The population considered was elderly (>65 years of age) patients with all types of SIF including bilateral sacral ala fractures. “Insufficiency fractures” were defined as those due to either low-energy trauma or no trauma at all. Studies of only young patients, high-energy injuries, and those outside the sacrum were excluded. Fractures secondary to malignancy were also considered ineligible. The control was conservative treatment of SIF (including bed rest, analgesics, and osteoporosis-treating medications) [4,14]. The interventions included sacroplasty and surgical fixation (including screw fixation, bars, and all forms of osteosynthesis). Cement-augmented screw fixation was included as a subset of screw fixation.  Some measure of treatment outcome was required for inclusion, for example, pain relief. Single-patient case reports, cadaveric studies, animal studies, and studies with no full-text availability in English were excluded.

MEDLINE/PubMed and Embase databases were searched by TS on January 6, 2022. Further snowball search of the references of eligible papers to identify additional studies for full-text review was done. Four separate searches of the literature were undertaken to identify studies that included at least two different treatment strategies. Their overviews are as follows: 1. Population (SIF) AND conservative AND sacroplasty AND screw fixation; 2. Population (SIF)  AND  conservative AND sacroplasty; 3. Population (SIF)  AND  conservative  AND screw fixation; 4. Population (SIF)  AND  sacroplasty AND screw fixation. 

**Figure 1:**
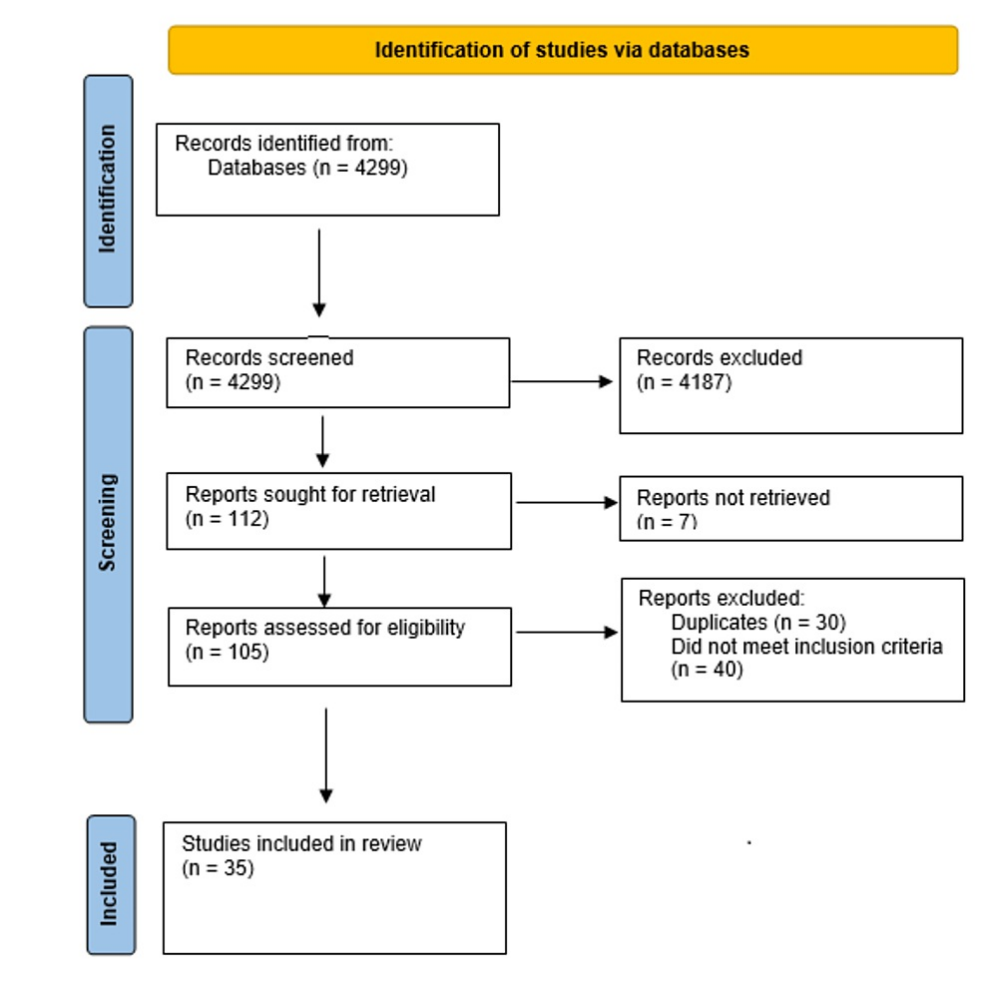
PRISMA flowchart showing the identified and included studies PRISMA: Preferred Reporting Items for Systematic Reviews and Meta-Analysis

Title and abstract screening were undertaken by two independent reviewers (PB and SK) manually and using Rayyan.ai (Rayyan Systems Inc., Cambridge, Massachusetts, United States), a systematic review online sorting tool. PB and SK did full-text review of the papers. Any disagreements were settled with discussion in the first instance followed by final arbitration by the senior author (MC-G). Figure 1 demonstrates this process. The following data were obtained and tabulated for all full-text articles reviewed: study author, date, pain (Visual Analogue Scale (VAS)) score (pre and post-intervention), nature of intervention, mobility, complications, duration of hospital stay, cost of treatment (where available), and mean follow up duration. Given that sample sizes were generally large (>50) for each treatment category, a decision was made to use parametric tests (mean, standard deviation, and unpaired T test). Online calculators, LibreText (April 11, 2022 update), and GraphPad [15] were used to do the mean, standard deviation, and unpaired T test calculations. 

Results

A total of 4299 studies were identified, of which 35 were eligible for inclusion; 11 cohort studies and 24 case series. The most consistently reported outcome measure among the studies was the pain score (VAS) pre- and post-intervention (Tables 1-2).  Reduction in VAS pain score was greater post sacroplasty (VAS difference 5.83, SD 1.14, n = 901) compared with conservative management (VAS difference 3.7, SD 2.71, n = 65) (p <0.0001) and after surgical fixation (with screws/rods +/- cement augmentation; VAS difference 4.1, SD 1.106, n 154) (p< 0.001). There was no significant difference between pain relief following screw fixation and conservative management (p = 0.1216). Upon subgroup analysis of the screw fixation group, the PMMA cement augmented patients (VAS difference 4.38, SD 0.79, n= 96) had better pain relief than those who had only screw fixation (VAS difference 3.63, SD 1.36, n 58) (p < 0.0001). 

**Table 1:** Pre- and post-intervention VAS scores for all included studies dV: change in VAS score pre/post intervention; ?: no VAS information provided in paper; Aug: cement augmented screw fixation; VAS: Visual Analogue Scale

Study	Number of sacroplasty patients	Pre-sacroplasty VAS score	Early post-sacroplasty VAS score	Late post-sacroplasty VAS score	dV sacroplasty	Number of conservative treatment patients	Pre-conservative treatment VAS score	Post-conservative treatment VAS score	dV conservative treatment	Number of surgical fixation patients	Pre-surgical fixation VAS score	Post-surgical fixation VAS score	dV surgical fixation
Rommens et al., 2015 [6]		VAS not measured											
Kortman et al., 2013 [4]	204	9.2	1.9		7.3								
Yang et al., 2018 [16] (+ balloon sacroplasty)	18	7.8	2.8	2.2	5								
Yang et al., 2018 [16] (- balloon sacroplasty)	27	7.7	2.9	2.6	4.8								
Wähnert et al., 2013 [17]										12	8.2 (Aug)	2.6(Aug)	5.6 (Aug)
Bayley et al., 2009 [2]	108	8.9	2.6		6.3								
Vanderschot et al., 2009 [18]										19	6.8	2.3	4.5
Kamel et al., 2009 [3]	19	8	4	1.6	4								
Schwetje et al., 2020 [7]		VAS not measured											
Andresen et al., 2017 [19]	40	8.6	2.5	2.0	6.1			8.6					
Höch et al., 2017 [20]										34	6.7 (Aug)	2.7 (Aug)	4 (Aug)
Choi et al., 2016 [21]	16	7.5	4.2	1.2	3.3								
Klingler et al., 2013 [22]	4	8.3	2.3	1.3	6								
Garnon et al. 2017 [12]										2	n/a	1.5	
Tsiridis et al., 2007 [11]						?	?	8		3	8	2	6
König et al., 2018 [23]										16	7.2 (Aug)	1.9 (Aug)	5.3 (Aug)
German Spine Congress Proceedings, 2012 [24]										5	9	3	6
Kortman and Ortiz, 2013 [4]	204	9.2	1.9			?	?	9.2					
Frey et al., 2017 [5]	210	8.29	3.63	0.66	4.63	34	7.49	1.22	6.27				
Onen et al., 2015 [25]	15	7.6	1.7		5.9								
Frey et al., 2008 [8]	40	8.1	3.4	0.8	4.7								
Lee et al., 2020 [26]	40	9.1	2.53		6.57								
Sandmann and Stöckle, 2018 [27]										8	7.6 (Aug)	2.5 (Aug)	5.1 (Aug)
Heo and Park, 2017 [28]	68	8.65	2.41		6.24								
Balling, 2019 [29]										26	8.8 (Aug)	5.3 (Aug)	3.5 (Aug)
Balling, 2019 [29]											9	5.4	
Hassan, 2015 [30]	8	9	3.4		5.6								
Mehling et al., 2012 [31]										11			
Hartensuer et al., 2020 [9]										448	?	?	
Talmadge et al., 2014 [14]	18	8.9	2.5	2.8	6.4								
Pulley et al., 2018 [32]										16			
Hopf et al., 2015 [10]						30	6.8	6.0	0.8	30	6.0	3.6	2.4
Andresen et al., 2021 [33]	1	9	2			1	7	4	3	1	7	2	5
Andresen et al., 2012a [34]	40	8.9	2.7	2.5	6.2								
Andresen et al., 2015 [1]	20	8.8	2.3	2.1	6.5	20	?	8.8					
Andresen et al., 2012b [35]	30	8.8	2.7	2.3	6.1								

**Table 2:** VAS summary VAS: Visual Analogue Scale score

	Pre-intervention VAS, mean (SD)	Post-intervention VAS, mean (SD)	Number of patients	VAS difference, mean (SD)	Percentage VAS difference
Sacroplasty	8.66 (0.47)	2.79 (0.68)	901	5.83 (1.14)	67%
Conservative	7.16 (0.34); Range 6.8-7.5	3.47 (2.39); Range 1.22-6	65	3.7 (2.71)	52%
Screw fixation only	6.64 (0.89)	3.01 (0.64)	58	3.63 (1.36)	54%
Augmented screw fixation	7.6 (0.87)	3.24 (1.28)	96	4.38 (0.79)	57%
All screw fixation (± augmentation)	7.25 (0.99)	3.15 (1.09)	154	4.1 (1.106)	56.5%

Twenty-one out of 35 included studies had follow-up information (Table 3). The most common follow-up period was 12 months, with a range from 20 weeks to over 15 years. 

**Table 3:** Follow-up duration

Author and year	Follow-up duration	Treatment
Klingler et al., 2013 [22]	20 weeks	Sacroplasty
Vanderschot et al., 2009 [18]	9 months	Screw fixation
Talmadge et al., 2014 [14]	48 weeks	Sacroplasty
Kamel et al., 2009 [3]	48 weeks	Sacroplasty
König et al., 2018 [23]	1 year (6 weeks VAS)	Screw fixation
Choi et al., 2016 [21]	1 year	Sacroplasty
Garnon et al., 2017 [12]	1 year	Screw fixation
Chandra et al., 2019 [36]	Most patients for 1 year	Sacroplasty
Pulley et al., 2018 [32]	1 year	Screw fixation
Andresen et al., 2012 [35]	1 year	Sacroplasty
Frey and DePalma, 2008 [8]	1 year	Sacroplasty
German Spine Congress Proceedings, 2012 [24]	1 year	Screw fixation
Tsiridis et al., 2007 [11]	1-2 years	Conservative, screw fixation
Mehling et al., 2012 [31]	14 months	Screw fixation
Andresen et al., 2017 [19]	18 months	Conservative, then Sacroplasty
Bayley et al., 2009 [2]	<2 years	Sacroplasty
Hopf et al., 2015 [10]	31 months	Conservative, Screw fixation
Myers et al., 2017 [5]	10 years, 2 years	Sacroplasty, Conservative
Heo and Park, 2017 [28]	15.86 years	Sacroplasty

Average total hospital stay duration was highest in surgical fixation (10.3±5.59 days) as compared with sacroplasty patients (4.1±3.9 days) (Table 4) and this was statistically significant (p < 0.0001). There was insufficient data to determine the average hospital stay duration for conservatively managed patients. Only one case series with three patients had information on the length of stay for a single patient who had conservative care (four days) [33]. After excluding studies involving an initial failure of conservative care prior to intervention, the length of stay remained shorter for sacroplasty patients compared with those who underwent surgical fixation (3±1.26 days vs 7.9±1.62 days) (p < 0.0001). 

**Table 4:** Duration of hospital stay Px: patient; Aug: augmentation; op: operation

Author and year	Hospital stay duration	Treatment	Number of Patients
Schwetje et al., 2020 [7]	17.1 days	Sacroplasty, Conservative	10
Höch et al,. 2017 [20]	14.1 days (9.6 days post-op).	Screw fixation	34
König et al., 2018 [23]	13 days	Screw fixation	16
Balling, 2019 [29]	9.3 days 9.6 days	Screw fixation	26
Hartensuer et al.. 2020 [9]	7-8 days Aug fix less stay than no Aug	Screw fixation	204
Tsiridis et al., 2007 [11]	Px 2: 1 week, Px 3: 5/7, Px 1: n/a	Screw fixation and Conservative	3
Hopf et al., 2015 [10]	23.7 days (pre and post-op)	Screw fixation and Conservative	40
Andresen et al., 2021 [33]	Px 1: 6 days, Px 2: 4 days, Px 3: 5 days	Screw fixation, Conservative, and Sacroplasty	3
Andresen et al., 2015 [1]	4 days	Sacroplasty and Conservative	20
Choi et al., 2016 [21]	5.5 days	Sacroplasty	16
Lee et al., 2020 [26]	1.66 days	Sacroplasty	40
Yang et al., 2018 [16]	2.83 days	Sacroplasty	45

Concerning improvement in patient mobility, there were no uniform modalities for measurement or assessment across the studies. Six studies related to screw fixation recorded mobility outcomes [9,10,17,27,29,32]. Wahnert et al. stated that all patients got back to pre-operative mobility but did not specify when [17]. A cohort where all patients mobilized pre-discharge but did not specify if mobility aids were required [29]. In another study, 52% of the patients mobilized with a frame, 29.73% with crutches, and 8.79% could not be mobilized before discharge [9] . Four studies related to sacroplasty reported mobility information [14,19,22,28]. There is clear short-term improvement in mobility post sacroplasty but studies with longer-term follow-up did not report on mobility. Two reported that all patients mobilized either on postoperative day one or were fully mobile at the time of discharge [1,22]. There was no clear mobility information for patients managed conservatively.

Discussion

There are a number of treatment options for sacral insufficiency fractures globally and there are no national guidelines in the United Kingdom to guide management. This systematic review set out to look for studies that had more than one modality of treatment of sacral insufficiency fracture in order to compare outcomes directly. However, most studies (n = 23) used only one modality of treatment. Of the remaining 12, five studies consisted of patients who had failed conservative therapy and went on to undergo either sacroplasty or surgical fixation [1,7,10,11,19]. One study compared patients who had surgical fixation with those who had cement-augmented surgical fixation [29]; another study compared sacroplasty delivered in two different ways (with or without balloon) [16]. One study compared two separate cohorts that were treated with either sacroplasty or conservative management [5]. One case series of three patients compared the outcomes of sacroplasty, screw fixation, and conservative management [33]. Three studies had no clear information about pain relief using the VAS score. A more recent retrospective study by Andreson et al. of 292 patients showed that patients with VAS pain score ≤ 5 benefited from conservative therapy measures; pain score of ≥ 5 was associated with significantly delayed mobility [34]. Mortality after 12 months was 21.7% for conservative, 8.4% for interventional, and 13.6% for the surgical fixation group, the differences being significant. Over 24 months, patients achieved the best independence after sacroplasty, followed by osteosynthesis and conservative measures. 

There was no significant difference between pain relief offered by surgical fixation versus conservative care. Of the 10 papers with information about surgical fixation, four had VAS scores taken at two separate time points post-opertively [10,11,23,24]. Most were followed up at 12 months postoperative with a further follow-up that showed further reduction in VAS at the second time (in one study) [20]. VAS score was better early postoperatively than later postoperatively. Five papers recorded VAS scores only once. The VAS is a widely known tool to measure pain ranging from 0 (no pain) to 10 (worst pain ever). This is the most reported measure of pain, hence its use in our analysis.

There were insufficient data in the studies reviewed about the duration of hospital stay in patients with conservative care. A prospective cohort study by Balling compared two groups of equal number of patients (26 each) who had either surgical fixation or cement-augmented surgical fixation [29]. Their pre- and post-intervention VAS average scores were similar. However, analysis of data from across all studies suggests that cement-augmented screw fixation gives better pain relief than screw fixation only. Though only one study was found with a direct comparison between sacroplasty and surgical fixation, statistical analysis of all results shows that sacroplasty was statistically significantly more effective (p <0.0001) than even augmented screw fixation in relieving pain. There was a shorter hospital stay (3-4.1 days) among sacroplasty patients compared to those who had surgical fixation (7.9-10.3 days) and this was also statistically significant (p<0.0001).

Due to the heterogeneity of reporting, the difference in the time taken to return to mobility between both treatment groups could not be ascertained. Information about short-term return to mobility was captured in only a few studies and there was no information about long-term mobility improvement. The most common complication of sacroplasty was the extravasation of cement, which occurred in 3.3% of cases. There was symptomatic (radicular pain) in two out of 485 patients (0.4%); one patient had surgical decompression and the other had nerve root block which led to the resolution of symptoms (Table 5). Most studies demonstrated no other complication. Potential complications include venous intravasation, which is often asymptomatic [37]. Regarding complications of surgical fixation, Balling [29] reported one instance of each of guide wire malposition (2%), screw pull-out (2%), haematoma (2%). König et al. reported one case (4%) of screw migration and another case (4%) of screw and washer migration into cortical iliac bone due to osteoporosis one year post procedure in their cohort of 20 patients [23]. These findings suggest sacroplasty may be associated with improved pain relief and fewer complications than surgical fixation. There are different methods of sacroplasty. Andresen et al. report no significant differences in outcomes between these [19].

**Table 5:** Reported cement leakage in included studies following sacroplasty

Study	Treatment	Study size	Number of cement leak	Outcome
Bayley et al., 2009 [2]	Sacroplasty	108	1	S1 nerve root pain, had nerve root block and pain resolved.
Höch et al., 2017 [20]	Augmented fixation	34	3	Asymptomatic
Choi et al., 2016 [21]	Sacroplasty	16	2	Asymptomatic
König et al., 2018 [23]	Sacroplasty	20	0	
Klingler et al., 2013 [22]	Sacroplasty	4	3	Asymptomatic
German Spine Congress Proceedings, 2012 [24]	Augmented fixation	5	0	
Kortman et al., 2013 [4]	Sacroplasty	204	1	Radicular pain. Had surgical decompression with good relief
Heo and Park, 2017 [28]	Sacroplasty	68	2	Asymptomatic
Balling, 2019 [29]	Augmented fixation	26	4	Asymptomatic
Sub-totals	Sacroplasty	420	8	1.9% cement leak; 0.5% symptomatic
	Augmented fixation	65	7	10.7% cement leak ; 0% symptomatic
TOTAL		485	16	Cement leakage: 3.3%, Symptomatic cement leak: 0.4%

There are a number of limitations with this systematic review. Most of the evidence was of levels 3 and 4. This comes with attendant selection bias, reporting bias, and bias in study design. There were insufficient direct comparisons between the treatment modalities, and data on conservative care outcomes were limited. In most of the studies with information about conservative care, the patients had tried conservative care as in-patients and failed, before having either sacroplasty or surgical fixation. Hence, it was difficult to get clear data on the duration of hospital stay of patients that had only conservative care. From the studies included, it is difficult to ascertain the proportion of patients who had conservative care and had very good outcomes (mobility, pain score), as most of these focused on patients with poor outcomes that had to have other treatments.  

## Conclusions

There is low-quality evidence concerning the superiority of treatment modality for SIF. However, available evidence from this systematic review points to sacroplasty being associated with better pain relief with significantly shorter hospital stay than screw fixation and conservative management. There is no clear information about the difference in mobility, cost, or complication profile between the treatment options. It is recommended that a randomized control trial looking at all three major options of treatment be undertaken with a minimum 12-month follow-up. This should assess pain score, mobility, functional scores, and cost of treatment. This would give clearer and higher quality evidence to help guide the establishment of national guidelines for the treatment of patients with sacral insufficiency fractures.
